# The Experiences of “Difficult Life” in Heart Valve Replaced Patients

**DOI:** 10.5812/ircmj.19147

**Published:** 2014-08-05

**Authors:** Mohsen Taghadosi, Robabeh Memarian, Fazlollah Ahmadi

**Affiliations:** 1 Department of Nursing, Faculty of Medical Sciences, Tarbiat Modares University, Tehran, IR Iran

**Keywords:** Heart Valve Prosthesis, Heart Valve Implantation, Qualitative Research, Life

## Abstract

**Background:**

Several reports, however, not comprehensive, have been presented about the experiences of patients with replaced heart-valve.

**Objectives:**

This study explores the experiences of the patients with heart valve replacement.

**Patients and Methods:**

A qualitative research with a content analysis approach was carried out on the patients with cardiac valve replacement during the year 2012 and 2013. A purposeful sampling using a semi-structured interview and open-ended questions (with the main question of "What problems did you have after the valve replacement?" followed by the exploratory questions) were performed until the data saturation. All interviews were recorded, then transcribed and typed. The data analysis was done according to Grancheim and Lundmen content analysis using the MAXQDA software.

**Results:**

Of 22 interviews taken from 13 participants, 430 codes were taken. Out of them, after deleting the similar items, 162 initial, 18 subcategory, and 5 subsidiary themes (problematic exposure with valve replacement, continuity of some difficulties, aggravation of life problems, insufficient support, and following a perceived care) were extracted. Based on the abstract and deep perception of the categories, the main theme of "difficult life" was explored.

**Conclusions:**

Aggravation and the continuity of some physical problems, and insufficient support after the valve replacement make the life difficult for the patients. Identification of these problems is necessary for planning and improvement of the patients' care, life quality, and survival through consultations, rehabilitation and education.

## 1. Background

In spite of significant advancements in science and technology, there are still a variety of physical and non-physical problems that man had little or no experience about them (1).

Each year, a large number of patients (9800 in America and 195000 outside America) undergo valve replacement (2, 3). Most valve replacements are performed because of the valve stenosis or insufficiencies (4). No report on the valve replacement is available in Iran; however, based on annual reports of 35 to 50 thousands cases for all heart surgeries, the first and the second rankings belong to coronary transplantation and heart valve replacements, respectively (5).

Although in general, the consultations must be carried out before any surgery (6), the number of valve replacement surgeries is increasing, which deserves the recipient's care and preservation (7). Considering the outcomes of valve replacement, prolonged and repetitive hospitalization of these patients need a long-life care (8). The right use of oral anticoagulants decreases the complications of thromboemboli significantly (9). The annual risk ratio of thromboemboli among the warfarin users and non-users are 1% and 4%, respectively (10). International normalized ratio (INR) for patients with mechanical aortic and mitral valve is 2-3 and 2.5-3.5, respectively (11-14). According to Menendez-Jandula et al. the permanent complications of valve replacement are as follows: thromboemboli, bleeding due to the anticoagulant drugs, prosthesis dysfunction, and the infectious endocarditis (15). The range of INR among the self-managed patients compared to conventional care is closer to the therapeutic range (16). More than half of the patients did not utilize the right dose of their drugs and had no information on the effect of the drug, its side effects, and the relevant life style (17). The outcomes of the insufficient caring system are bleeding, recurrent thrombosis, valvular insufficiency and death (18). The injury-inducing experiences of these patients are different and rang from the time of diagnosis and need for replacement to taking the decision for operation (7, 19).

In a report from India, the rate of premature delivery, neonatal weight loss, mortality of mothers and an “Apgar score ≤ 8” was higher among the cardiac-valve replaced patients (20). The physical problems and complications before and after the operation are serious (21). The rate of atrial fibrillation is 30%-40%, which results in thromboemboli, low cardiac output, and higher mortality and morbidity (22). Most of the valve replaced patients had changes in daily physical activity, but in the trained patients, the problems of bleeding, dyspnea, sleep disorders and the decrease in activity tolerance was significantly less (23). In another study on 320 patients, it was seen that the majority of them had bleeding, ecchymosis, also one-third of them had drug interaction in their prescription (24). From the patients’ perspective, the determinations of their needs are considered a necessity for providing nursing care (25). The patient's experiences are important for professional nurses, and the cultural background could influence the individual's reaction and their caring follow-ups (26, 27). The available reports denote an unfavorable caring condition. As the different social and cultural backgrounds affect the care and problems of the patients with cardiac valve replacement, and no comprehensive study has done for such patients, the explanation of a caring program is necessary.

## 2. Objectives

This study aims to explore the experiences of cardiac valve replaced patients in Iran in order to find a better plan for their care.

## 3. Patients and Methods

For exploring the perception and experiences of mechanical valve replaced patients, we used the content analysis method. The research setting was the educational hospitals in Tehran and Kashan. The participants were enrolled by purposeful and expert sampling with maximum variation. The analysis taken from every interview was a guide for the next interviews, and the sampling was done until the saturation of data. The selection criteria for participants were purposeful, and the patients were selected based on the experiences of living with mechanical valve. Insufficient experience in any participant was considered as the exclusion criterion. The interviews took place in the therapeutic centers. The permission for study in clinical settings was issued by the Ethics Committee of Tarbiat Modares University (TMU). 

The study was done based on informed consent, privacy and the validity of the study. Before the interview, the purpose of the study and also the privacy of the recorded interviews were described. Then in the light of willing to participate, the interview started with an open-ended question on the experiences of life with a prosthetic valve. Afterwards, the interview was followed by exploratory questions (e.g. please explain more and give an example to clarify it). The average time of each interview was 57 minutes. The interviewer was a qualified person in terms of having the experience of working with such patients (≈ 21 years) in the critical care units of educational hospitals. He is also running his dissertation in this field right now.

The simultaneous analysis of the interviews was performed using the Lundmen and Grancheim content analysis method in five stages (28). In this study following the completion of every interview, its content was written and typed. For a general understanding of the patients’ words with respect to the research goals, the interview scripts were read several times. Then, (using the MAXQDA software) to provide the validity and reliability of the results, we acquired 4 criteria of creditability, transferability, dependability and conformability (29). Prolonged engagement in the field from September 2012 to March 2013 helped us to establish some trust and support with the participants, providing an opportunity to collect the data. To make sure that the analysis reveals the patients’ experiences, member checking was performed during the data collection, and where needed, some changes were done. For the creditability of the findings, we tried to present the quotes of participants with fidelity, so that the readers have a better judgment over the results of the study. To confirm dependability and conformability of the data, the interviews, and the results of the analyses, like the initial codes, and subcategories were audited by some experts. We implemented the external check method by using two authors (the first and second author) expertise in health education and then peer-checked by two PhD students in health education. Furthermore, maximum variation of sampling confirmed the transferability of data. Sampling strategies allowed for maximum variation to occur and a vast range of views and perspectives to be considered.

This project was approved in the Ethics Committee of TMU under the code: D52.2156, July, 2013 with the consideration of the above-mentioned guidelines.

## 4. Results

Twenty-two interviews with 13 participants were carried out (Table 1). From the deep and rich explanations of the participants, 430 initial codes were derived among which after deletion of the repetitive codes, 162 initial codes were remained. After several reviewing and summarizing and based on similarities and differences, the codes were classified into 18 categories. Based on the conceptual and abstract nature of their meaning, the essence of the codes were nominated and identified in 5 themes using the analysis and comparisons. Ultimately, a common theme of "difficult life" was derived.

**Table 1.** Demographic Features of the Participants

Variable	Number
**Gender**	
Male	5
Female	8
**Mean Age, y**	48.7
**Marital status**	
Married	11
Widow	1
Single	1
**Education level**	
Illiterate	1
Elementary School	8
high School	4
**Type of prosthetic valve**	
Mitral	9
Aortic	2
Both	2
**Occupation**	
Retired	2
Housewife	7 (all women)
Farmer	1
Worker	1
**Time after replacement**	2 wk - 27 y
**Interview length**	45-65 min

### 4.1. Difficult Confrontation With Valve Replacement

During the time of valve replacement, these patients are confronted with a critical crisis, including facing with a difficult condition, the painful tolerance of previous care, and the aggravating factors. For years, these patients were living with stenotic or insufficient valves, and many procedures were performed on them for diagnosis and treatment. Their problems were aggravated with the advancement of the disease. Sometimes, there are disagreements between physicians for performing surgery. Some physicians prefer to postpone the valve replacement for some reasons, e.g. warfarin use. These hard and non-tolerable conditions are unfavorable experiences for the patients. The postoperative warfarin use and its interaction with other drugs cause many problems for diagnostic procedures. Most therapeutic centers do not perform such procedures because of the specific and complex conditions of the patients. These problems by themselves cause problems in patients' lives.

"My esophagus echo was done, and now I have to do it again. My mind was occupied all the time with echoing. It is hard for me. I must go to Tehran for doing this. My pervious echo bothered me too. I am afraid of having the same experiences." (participant # 12, female, 34 years old, replacement of both valves).

"For angiography I was there for 45 minutes. I was so sick that day and it was a hard time. I felt a severe palpitation. They gave me no anesthesia. Doctor instructed me to take a deep breath." (participant # 7, female, 67 years old, mitral valve replacement).

Doing diagnostic and therapeutic procedures for these patients are inevitable. These procedures are unfavorable experiences and make the patients to avoid their test repetitions. Sometimes, despite the push and emphasis by the physicians for the treatment, the patients tolerate the problems and postpone the surgery.

### 4.2. Continuity of Some Heart Problems in Patients With Prosthetic Valve

These problems consist of the continuity and aggravation of the cardiac signs, difficult access to a sustainable care, lack of the tolerance for associated problems, and difficulties of having a conventional life. During their lives, most patients often have bleedings, thrombosis and infections followed by difficulties due to changing in the dose of the drug. The routine life of valve replaced patients is influenced with the rheumatoid and valvular diseases since the young ages. Because of these conditions, the patients are facing with many limitations in their interactions with others.

In addition, after the valve replacement, they will have multiple problems such as taking medications, disregarding the diet/drug regimen, or forgetting the drug. In females, the obstetric and gynecologic problems also added to their problems that need some support. The necessity of repeated INR experiment, repeated problems of the patients and the heavy expenses, repeated referring to the centers, low income, insufficient support and the tolerance of a difficult condition exhaust the patients.

"After the operation, I came to home with a severe fever and chilling. I had to go to the hospital in Tehran again. There were some leak and infection around my valve. I was hospitalized 2 months there and was operated again“ (participant # 1, female, 40 years old, aortic valve replacement). These patients are suffering from cardiac problems due to the changes in the size and structure of the heart (e.g. palpitation or heart rate alteration).

"The tick-tick sound of the valve bothers me. The sound is unfamiliar to me. I cannot sleep because of the heart sound. I feel heaviness and discomfort in my chest. I use my drugs, but they have no effect on the sound. On standing it beats faster and signals me to take a rest. I couldn't sleep” (participant # 3, female, 32 years old, and mitral valve replacement).

Valve replacement and change in the blood flow path could take a long time for a patient to be adapted to. In addition, in the case of the disorders in other valves, patients have weakness, fatigue, and dyspnea, which are related to their valve disease.

"This six-month period was a hard time for me. During that time, I was unable to lift a 3-kg weight. When I did a task, it bothered me for several days" (participant # 8, male, 50 years old, both mitral and aortic valve replacement). The physicians limit their therapeutic and diagnostic procedures (e.g. dental services) due to the risk of bacteremia. These conditions suffer the patients.

"Most of my teeth have carries; I wish I had treated them before the operation. Now no physician accepts the responsibility of their treatment. Sometimes, I brush them, but in that case, it also causes bleeding" (participant # 12, male, 34 years old, mitral and aortic valve replacement). These patients tolerate the unfavorable conditions that deserved to be familiarized with and got more attention.

### 4.3. Aggravation of the Problems of Life

Pregnancy and high-risk delivery, worry of the transmission to the children, negative thinking due to observing unsuccessful experiences are factors that aggravate the problems of life. Patients usually face with special problems during their lives (especially during the pregnancy and delivery).

Because of the necessity of the continual use of anticoagulants and monitoring as well as changing the anticoagulant regimen at the beginning and the end of the pregnancy, patients need support from the therapeutic team. Otherwise, their lives get harder and both mother and fetus are confronted with more problems.

"The hardest thing for me was the PT control. I had bleeding in my kidney at 7 months of age. I was hospitalized, and it took me 1 week to adjust my PT and stop the bleeding" (participant # 13, female, 45 years old, mitral and aortic valve replacement). 

"After the pregnancy when I showed the lab reports to my doctor, he got agitated and said: You made me crazy. Your heart's problem was not enough to tolerate. You are pregnant" (participant # 4, female, 40 years old, mitral valve replacement).

Sometimes both patient and physician are informed of the pregnancy late; the patients do not have enough information or become aware of the precautions. For example, they might use the teratogenic drugs for a long time that can be followed by non-compensable complications for both fetus and mother.

### 4.4. Insufficient Support

Some factors (e.g. carelessness regarding the education on discharging from hospital, limited support regarding rehabilitation of the valve replaced patients, or incorrect rehabilitation of patients) are among the evidence that indicate the valve replaced patients are not supported and do not receive regular care program and necessary education to prepare for a life after the valve replacement. 

"I was always waiting for the doctors and nurses to instruct and educate me on my care." (participant # 2, female, 47 years old, mitral valve replacement twice). 

The caring condition is not stable. It is partly due to the care team; the working shift of the physicians changes constantly, and they do not care for the needs of patients. The patients have to refer multiple times for having access to their physicians and also doing the necessary tasks.

"The origin of all my problems is the shortage of enough information and my forgetfulness. Nobody educated me in this regard. I followed the care precautions by hearing and asking from others. If there was a program in transferring the experiences of valve replaced patients to those undergoing the operation, it would be so efficient" (participant # 5, male, 55 years old, mitral valve replacement).

The patients do not often receive rehabilitation due to insufficient financial support. If they receive some rehabilitation, it would be based on the exercise only and the actual needs of the patients are disregarded. The patients only benefit from periodic visits of the physicians and are faced with critical problems due to the discrepancy between the physicians, shortage of time, forgetfulness and the failure.

"I wish my doctor had checked my teeth before the operation, otherwise I would have not been hospitalized for them” (participant # 8, male, 50 years old, mitral and aortic valve replacement).

The unavailability of caring system and insufficient support causes the patients encounter with multiple problems during their lives.

"No medical center takes the responsibility of my teeth problems. They left them untreated and sent me to other centers" (participant # 6, female, 73 years old, both mitral and aortic valve replacement). 

All these instances signify the need for sufficient support after the valve replacement.

### 4.5. Following the Perceived Care

In some instances (e.g. the permanent control of coagulatory tests, long-term use of drugs, permanent fluctuations of INR, sudden occurrence of complications, and long-term use of drugs) the valve replaced patients have to follow the care program after the replacement. Regular and constant use of drugs, drugs interaction, doubt and forgetting the drug use all can be dangerous and divesting. Sometimes the patients were referred for vaccination and got ready for following the preventive precautions for the reduction of infection risk of the valves. The drugs have interactions with warfarin, and the patients face with a vicious cycle of problems. "Until now, I used antibiotics for my urinary infection; each time I had an abnormal PT, I had to discontinue my drug" (participant # 7, female, 67 years old, mitral valve replacement).

"For my osteoporosis, the doctor prescribed calcium. It changed my PT. It caused bleeding, and I had to discontinue the calcium and tolerate the osteoporosis" (participant # 4, female, 40 years old, mitral valve replacement).

Another problem of the participants was changing the dose of the INR, and warfarin during the travel and different seasons of the year. "Weather condition affects my PT. In winters, I take more warfarin, but in summers, I take less" (participant # 13, female, 45 years old, mitral valve replacement).

Following the warfarin use the patients are facing with bleeding and anemia due to the aggravation of the bleeding. "Sometimes, I have bleedings in some parts of my body; the bleeding sites get blue. I asked the reasons for these; my physician said that it is due to warfarin" (participant # 7, female, 67 year, mitral valve replacement).

Experiencing these problems incurs a lot of time and heavy expenses on the patients and causes multiple problems for them. This evidence reveals the necessity for the support of the valve replaced patients.

## 5. Discussion

Heart valve replacement is one of the successful treatments for valvular disorders. However, findings of the present study reveal the common theme of “difficult life” and signify the real experience, and perception of valve replaced patients. Analysis of the experiences of the participants indicates that the lives of the patients continue with difficulties. Also, their problems originate from the following sources: bad confrontation with valve replacement, difficult life with prosthetic valve, intensifying the life problems, and insufficient support following the perceived care.

Parts of these problems are due to the condition and facilities of the operation or insufficient and unsuitable distribution personnel for quality care. In addition, to access this kind of care, the patients tolerate a lot of costs and time. For their care, patients do not receive necessary education. Patients’ participation is an important factor in anticoagulant therapy, and its careful follow-up is critical for the prevention of thromboembolic events (3). Patients with mechanical prosthesis require permanent anticoagulant medication which in the case of incorrect use precipitates the risk of the bleeding and thromboembolic conditions (e.g. sickness, pregnancy, surgery and drug use) (4). The discharge plan in hospitals is not suitable and the patients do not follow the discharge education plan.

 These conditions result in noncompensable failures. Bloomfield reported that 24% of heparin users in their first trimester, had the valve thrombosis (30). During pregnancy hypercoagulation of blood increases the risk of valvular thrombosis (31). The warfarin dose of more than 5 mg during the first trimester could result in multiple anomalies and failure to thrive (32, 33). In addition, the heparin use during pregnancy is followed by the increased risk of thromboemboli and valve dysfunction (34).

After coronary surgery, heart valve replacement is the second routine surgery in Iran (5). However, most patients after the operation are discharged with no education and suitable follow-up plan. Because of changes in life condition, food and drug regimen and travel, patients are facing with multiple problems signifying the prominent role of nurses in relieving the problems. Menendez-Jandula et al. revealed that the group education reduced the mortality and complications (15). The patients with mechanical valve during their lives need the care and education of nurses and when they do not receive it, they will confront with multiple problems. The Holroyd's study revealed that the most prevalent problems of Chinese patients ranged from delay in on-time treatment to the continued bedside care (35). The successful treatment of patients depends on different factors, like post-discharge consultation, which in turn decreases the costs (30). The on-discharge education, especially the programmed education, could result in receiving more information, decreasing the anxiety, improving the self-care after discharge, accelerating the recovery process and satisfying the patient (36).

The physical problems of the patients after valve replacement decrease; however, bad confrontations with the surgery, continual progress of the problems, insufficient support and merely following the perceived care make the life troublesome for them. Identification of these problems helps to plan and reconstruct their care through support, consultation, education and rehabilitation, which finally promote the survival and quality of life for these patients.
